# The Why and the Wherefore, or the Philosophy of Life, Health, and Disease

**Published:** 1846-10-01

**Authors:** 


I. The Why and the Wherefore, or the Philosophy of Life, Health, and Disease. By *Charles Searle*, M.D. M.R.C.S.E. 8vo. pp. 266. Churchill: London, 1846.

II. Liebig's Physiology applied in the Treatment of Functional Derangement and Organic Disease. Part I. By *John Leeson*. 8vo. pp. 216. Renshaw: London, 1846.

III. Homœopathy, Allopathy, and Young Physic. By *John Forbes*, M.D. F.R.S. 12mo. pp. 121. Philadelph. Edit. 1846.

We place the titles of these publications together at the head of the present article, not because they treat of any subject in common, or resemble each other in either object or execution; but inasmuch as each of them, although in very different degrees, furnishes us with the opportunity of animadverting upon the tendency, by no means rare at the present time among medical writers, to pay undue homage to the advantages derivable from various forms of quackery, and to most unfairly depreciate those which the legitimate practice of physic has at its command.

Charlatanism always has existed, and probably always will prevail in the world; and certainly, in spite of our advances in a *quasi* education, which furnishes the learner with the means of perusing without the power of judging of the value of the lucubrations of pretenders, at no period of time has it ever been, professional and non-professional, more rife than at the present. One remarkable feature in the history of its modern progress as distinguished from that of former times deserves our especial notice. Heretofore, quackery received no favours, or even quarter, at the hands of the regular members of the profession. In fact these, in the shape of the various authorities in the different parts of Europe, as Faculties, Universities, Colleges, &c., prosecuted an open war against quacks with unrelenting vigour and energy. We do not say that such proceedings did not occasionally lack discretion; for, by their indiscriminate, and therefore sometimes unjust and vindictive character, they often had the mischievous effect of arousing public sympathy in favour of the proscribed, furnishing a notoriety which was the one thing desired. The very fact of the existence of such errors, however, proves the utter absence of connivance at, or encouragement of, these irregular proceedings on the parts of those who had been enrolled members of an important and highly responsible profession. How stands this matter at the present time? Our incorporated medical bodies, hypothetically supposed to represent the interests, the honour, and the dignity of our profession, neither possess, nor have shewn themselves desirous of possessing, the requisite power for the suppression of quacks and impostors of any kind, however flagrantly these may invade the rights of educated practitioners, or however widely they may spread abroad their desolating mischief amid the community. More than this: they do not even reprove, discountenance, or expel any of their own members who encourage, or even take part in, any of the impostures of the age.

But, besides this apathy and indirect encouragement thus offered to impostors by the corporate bodies, various individual members of the profession have, from time to time, by act, word, or book, added not a little to such encouragement, and to the perpetuation of the delusions prevailing in the public mind upon the subject. There is, perhaps, scarcely a quack medicine in existence which has not at one time or other been prescribed by members of the faculty, and, *proh pudor!* sold by others of them; while each of the arch-quackeries of the present day, whether it be mesmerism, homœopathy, or hydropathy, has received open encouragement from members of the profession, whose acquirements and prior position taught us to expect better things at their hands. And, whether it arises from the contagious influence of a few great names, the involvement of the medical mind in, or its submission to, the fashionable delusions of the day, or its easy gullibility by specious appearances and unauthenticated facts, we know not: but certain it is that it is as common as possible to find medical writers furnishing their more or less complete approval of certain forms of quackery, and deprecating the treatment which is pursued by the *non-illuminati*. For, let it be observed that, approval of the new schools and abuse of the old one, proceed *pari passu*, and that eloquent tirades upon the evils inflicted by the demon of polypharmacy, and cutting sarcasms upon the presumptuous and dangerous individuals who would fain endeavour, by means of some poisonous, horrid, filthy drug, given too in imaginable quantities, to cut short the course of a disease which should have been left to Nature's all restorative power, are standing topics of discourse with this class of writers. The works we have in hand contain specimens of this injudicious procedure, one or two of which we proceed to exhibit. Dr. Searle, after stating what he considers to be the two principles of the operation of *hydropathy*, [viz. 1, the purification of the blood and the reduction of præternatural excitement—and 2, the exciting and giving tone to the skin, and thus invigorating the entire system] goes on to say that, he considers it a most useful practice in gout, rheumatism, scrofula, various cutaneous diseases, dyspepsia, general debility, &c.; and adds,

"Although open to some objections, it assuredly embodies principles which, if judiciously carried out, are of most useful application in the treatment, not only of the large class of affections previously adverted to, but many others also; inasmuch as it, in an eminent degree, develops and augments all the natural powers of the system—and these are the only curative means in any case. The hydropathic practice, it will be well to observe, embodies in it the system of the trainer. * * * * * * * * The system of Preissnitz, I am however bound to say, does more than this, as it includes, in addition to active exercise in the open air, the mind's repose by abstaining from all active mental employ: with abstinence also from all warm fluids and exciting beverages, a plain, wholesome diet of meat, bread, vegetables, fruit, and cold water, being only allowed, with early rising, and early return to bed; and last, though not least in amount of beneficial consequences, preserving the blood from contamination and further deterioration, by excluding physic altogether! or when the blood is so contaminated, or by other cause deteriorated, as we have previously spoken of as being the case in gout and many other affections, purifying and divesting it thereof, by washing out the impurity with cold water, and expelling it from the system through the agency of the skin and the kidneys.

"This system, I must avow in conclusion, embodying as it assuredly does, if not all the requirements of a complete system of treating disease, yet including so many pre-eminently useful principles, deserves most undoubtedly great commendation: and it is not only puerile, but discreditable, to say in disparagement of its author, although but a peasant, that he has taught nothing that was not known before: inasmuch as it is not what may be learnt, but what is practised by man, that constitutes desert." P. 264.

Mr. Leeson thus favours us with his opinion upon the abuse of drugs in this country, and the advantages of *homœopathy.*

"The indiscriminate employment of drugs of the most offensive and drastic kind, of mineral compounds of the most poisonous description, and of the metallic salts, irritating and violent in their action, by the old and by many of our modern practitioners in medicine and surgery, often led, as they do now, to the most disastrous results: while those actions upon the body, the result of surrounding influences, and the principles of restoration by nutrition, and through the science of the chemical forces, were entirely unknown to them, or not respected sufficiently to be subjects for their consideration. These systems of treatment in themselves were sufficient to derange the healthy structure, or to convert simple cases into dangerous maladies.

"Wounds, bruises, ulcers, glandular irritation, inflamed textures, amputated surfaces, with all other such cases, were greatly aggravated, if not brought on to fatal results, by such treatment. The homœopathic or do-nothing system, or the plan of letting diseases take care of themselves, would gain for the charlatan or impostor the highest reputation; whilst the character of the legitimate practitioner would sink in public estimation, were he to pursue the plans which are often taught him under the direction of his Halls and Colleges.

"There are about 410 preparations in the Pharmacopœia of the Royal College of Physicians, which no doubt are considered by that learned body useful for medicinal purposes, or else they would not have been retained there. It is from this collection of preparations that the medical youths of this country are instructed to cull their remedies and apply them in the treatment of every form of disease. Now, any practical man, of 10 or 20 years' standing, must have found that 400 of these preparations are of little or no value whatever in the treatment of any form of disease, and that about the remaining 10 might have assisted him in reducing, at one time or other, cases occurring in every department of his practice. * * * * * * Hahnemann might have been considered a great modern improver in the art of constitutional treatment, had he not surrounded his do-nothing system with the most extravagant absurdities —had he not left acute inflammatory disease to the risk of homœopathic treatment: in other respects, his system often obtained great success in cases which received no advantages when under the medical treatment of some of our most *eminent and experienced practitioners.* Homœopathy has therefore its merits and demerits; and if the shrewd German had not something in his invention, he would not have received the sanction of one of our most distinguished modern philosophers." P. 9-12.

These, however, are mere incidental observations, the one appearing in a work containing several good things, and the other prefacing a worthless book of no weight or authority whatever. Dr. Forbes' publication is a laboured production, written with the distinct view and object of indicating the necessity and approach of a "Reformation" in the practice of physic—Hahnemann acting the Luther for the occasion. From the position of its author as a medical reviewer and court physician, and his reputation as an exposer of mesmerism, apparently so anxiously sought for, the tone he adopts, the admissions he makes, the suggestions he offers, and the blunders he commits, may do great harm, if not protested against, as not exhibiting the creed or the feelings of the profession in general.

Our limited space forbids our following his arguments and illustrations *seriatim*, and will allow of our only adverting to some of the most important conclusions he arrives at. It results from his examination of the claims of the Homœopathists, as far as the limited and imperfect data published by Fleischmann and Henderson permit it to be made, that he allows about an equal degree of curability of disease may be achieved by the homœopathic as by the allopathic mode of treatment; and as the whole course of his prior reasoning goes to show that what the homœopathists attribute to the operation of their infinitismal doses of medicine, is really brought about by the agency of nature, it follows that if diseases were left to their natural cures these would be about as numerous as they are at present, under the operation of the ordinary mode of managing them. It will be observed that this important conclusion, and the reformation to be based upon it, depend entirely upon the accuracy of the data furnished by the homœopathists. Now, in the first place, these are admitted by the author as being frequently so imperfect in their particularizations, as to render them worthless or dangerous for statistical purposes; and in the next, however illiberal it may seem, we are disposed to place much less faith in the veracity of facts which have been compiled and published for the specific purpose of propagating a new system, than if no such object had been held in view during their accumulation. Observation of human nature shews us that, when a new doctrine has to be promulgated, facts or pretended facts, are but too easy an acquisition; and certainly we are not prepared to believe that the *morale* of those who have deserted their profession of faith and are anxious to find any excuse for such desertion, is in so satisfactory a condition as to warrant us in supposing that falsification of statements, by the concealment of unsuccessful cases and an unfaithful relation of such as recover, is an impossibility or even improbability. Where experiments have been conducted by the homœopathists themselves in the hospitals of France and Germany, but under the supervision of men accustomed to observe with exactness and record with faithfulness, the results have been found to be entirely adverse to their cause—and that in countries accustomed to the observation of the effects of the treatment of disease by expectation. Experiments of this kind have not, as far as we are aware, yet been made in our own hospitals, and it is to be hoped that such will never be permitted: for we believe the poor creatures who, with confidence or through necessity, are entrusted to our care, are not those upon whom rash statements or blameable curiosity should allow us to put such trials into force. There is an abundance of voluntary dupes who deserve neither commiseration or mercy; and we suppose the practice of most of our readers must have acquainted them with instances in which the folly of such has met with its due reward.

Dr. Forbes thus states the inferences which may be drawn from the consideration of the homœopathists' alleged facts.

"1. That in a large proportion of the cases treated by allopathic physicians, the disease is cured by nature, and not by them. 2. That in a lesser, but still not a small proportion, the disease is cured by nature, in spite of them; in other words, their interference opposing, instead of assisting, the cure. 3. That, consequently, in a considerable proportion of diseases, it would fare as well, or better, with patients, in the actual condition of the medical art, as more generally practised, if all remedies, at least all active remedies, especially drugs, were abandoned. We repeat our readiness to admit these inferences as just, and to abide by the consequences of their adoption. We believe they are true. We grieve sincerely to believe them to be so; but so believing, their rejection is no longer in our power; we must receive them as facts, until they are proved not to be so."

In illustration of Nature's agency in accomplishing what homœopathist and allopathist claim as due to their own special instrumentality, Dr. Forbes enumerates several circumstances, such as the results derived from expectant medicine, from quack-medicines, from mesmerism, from influencing the imagination, from diet, hydropathy, &c. With the exception of quack-medicines, we admit that all these are citable in proof of many diseases being curable without the aid of *drugs;* but it is exhibiting but a poor degree of knowledge of the actual condition of the practice of medicine among the mass of practitioners of this country, to endeavour to make it be believed that they place the whole, or in many cases, their principal, reliance upon drugs. To them the value of diet, change of air, the various hygienic appliances, the necessity of, in many cases, abstaining from medicine, and in many others of not relying upon it as their principal means, are as well known as to their critic. The fact is there are more ways than one of curing the same disorder. The patient who has plenty of time, whose confidence is not easily shaken, and whose purse will admit of the delay, may frequently be enabled to exchange the disagreeabilities of drug-taking for the quietude of expectancy, or the pleasures of change of air, scene, mode of life, &c. But for the man whose very *status* for years to come may depend upon whether his illness is one of days or weeks, whose *morale* is depressed and despondent by its prolongation, it becomes a mercy and a benevolence to quicken the actions of his system, and eliminate the cause of its disorder, by a more rapid process than nature usually employs, be this by a drug ever so nauseous. And that this frequently can be done, who does not know? The slow-coach business is well enough for the wealthy, but is destructive to the poor. How the fact of quack-medicines having occasionally worked cures can be drawn into a proof of the general inefficiency of drugs, we cannot see. It is well-known that many of them consist of anything but inert substances, so that alarming consequences, and even death itself, have not unfrequently resulted from their employment. Taken as they are by such multitudes of persons, it of course must happen that occasionally they effect a cure, and more frequently give relief, or what is mistaken for such. The ground of opposition to their employment is the absence of adaptation to the special exigencies of the case, not their supposed inertness. That would only be an affair of the pocket. When Dr. Forbes, too, cites the effects of the Imagination, Mesmerism, and the like, as proofs of the efficacy of the do-nothing policy, we also feel obliged to dissent from his views. The influencing the imagination has always been, and always will be, a recognised, and sometimes a powerful, influence in the hands of the legitimate practitioner. Who is not aware that one of the most essential steps for the acquisition of dominion over the disease is the securing the confidence of the patient as to his attendant's power of treating it? May not a disease be treated by precisely the same means, but with quite different results, by two different practitioners? What man of sense ever relied upon drugs alone for the management of a long and tedious disease, to the neglect of the agency of that balm of life, hope? We will even give the homœopathists, mesmerists and *hoc genus omne*, the credit of producing their cures, when they do produce them, through the agency which the means they employ exerts upon the minds of the patients, and may contrast this advantageously with the system of expectation in which Nature is left to fight her battle, deprived of one of her most puissant allies. That such means will thus effect a cure in certain classes of disease we have no intention of denying, although we may be asked, why then do we object to their admission amid our other ordinary therapeutical resources? We reply, that their elevation into a *general system* for the treatment of disease is what we chiefly object to, as, while we acknowledge that they may be occasionally serviceable in one class of diseases, we are far more certain of their hurtfulness in another; and we cannot consent, for the sake of the advantage which might in some few cases be derived from their employment, but which might often as well be obtained from other measures unassociated with the evils which attach to them, to give that sanction to a system of quackery which would inevitably be afforded even by their partial employment by the profession. It may be said that the means which, in the hands of the present possessors is neither more or less, from the general and indiscriminate nature of its pretensions, than gross quackery, might become, through its intelligent employment by professional men, at least a valuable adjuvant in appropriate cases; and why, therefore, discourage the partial approvals which are ever and anon appearing in medical works, or at least that class of medical works which are intended as much (or more) for the public as for the profession. We reply that it is derogatory to the profession, and injurious to the public for regularly educated medical men to enter into the same career, however modified, with quacks and impostors. When once a medical man begins dabbling in hydropathy, mesmerism, homœopathy, &c., he runs the risk of becoming confounded with the herd of adventurers who are only eager to take advantage of the popular delusion of the day, and must be content to lose caste in the eyes of his professional brethren accordingly. We do not say that, on the failure of the means which the legitimate practice of his profession places at his disposal, he would not be justified in employing such as accident, or even the proceedings of quacks or imposters, may have acquainted him with. But these are rare cases, and not to be confounded with that morbid desire which seems to seize some professional persons, of patronizing every novelty, however absurd its pretensions, and questionable its promoters. Were things ordered as they should be, no man who had not received a medical education would be allowed to experiment upon the lives, health, and safety of the public, as he may at present, providing only he starts some new and preposterous humbug; and then each proposal for the relief of human ills would be submitted to the ordeal most competent to judge of its merit, and if any good were proved to reside therein, it would be appropriated to the special cases calling for its employment. At present, while an unbridled charlatanism envelops everything, the legitimate practitioner may flatter himself he rigorously limits his approbation of these various procedures to the cases for which they are adapted; but, in point of fact, his approval is heralded abroad, his limitations disregarded, and the public delusions only confirmed by one who should have done his part in their dissipation. Thus we always have, and always shall, set our faces against professional works addressed to an ignorant public in praise, however qualified, of measures which, whatever good they may contain, are indisputably in the hands of quacks and adventurers of all kinds. Writers are seldom sufficiently alive to the responsibilities they incur by the publication even of what they may consider the truth!

Upon this last point Dr. Forbes more than once emphatically states his opinion. Thus, he says in one place—

> "But many of our readers, we expect, will be of opinion that, in admitting what we have done, we are betraying the cause of legitimate medicine, and lending our aid to extend the heresy of homœopathy. If such should be the results of our admissions we cannot help it. We have said only what we believe to be true: and if what we believe is in reality the truth, the promulgation of it cannot lead to evil. Truth is good. If the art of medicine, as we profess and practise it, cannot bear investigation, and shrinks before the light of truth, from whatsoever quarter it may come, it is high time that it should cease to be sanctioned and upheld by philosophers and honest men. If, on the contrary, it be true and good—even if it be only but partially true and moderately good—the stirring touch of inquiry and the stimulus of opposition cannot fail to benefit it in the end."

No one will dispute the abstract proposition that "Truth is good:" but then comes the question, supposing that Dr. Forbes has stated but the truth, whether he has chosen an appropriate time for its promulgation. We have already stated that he has based his whole argument upon an insufficient foundation, and has very much exaggerated the case as against the allopathic mode of treating disease; and we think he is no less unfortunate in the reason he assigns as the immediate cause of publication. It seems that the subject has long occupied his thoughts, but that these have been at last forced from him prematurely by the fact of the conversion of Professor Henderson by the homœopathists. Without attaching so much importance to the secession of a very second-rate man, as Dr. Forbes seems to have done, we look upon it, as far as it goes, in the light of an evil, and as such it ought to have impressed additional cautiousness upon those who had the opportunity of expressing an opinion regarding it, and certainly not have been seized upon as an occasion for bearing testimony in favour of the practical advantages derivable from homœopathy, however erroneous its doctrines might be. Any opinions of this kind, although appearing at first in a professional organ, were certain eventually of reaching a general circulation, and should therefore have been considered with as great carefulness, as if popular and unintelligent perusal were the necessary consequence of their delivery. We look upon the present period as a singularly inappropriate one for exposing the condition of our profession to profane and mocking eyes, and exaggerating its errors, its shortcomings, and its excesses, for two reasons in particular. In the first place, perhaps the public mind was never so much occupied with various pseudo-professional quackeries as at present, and never so disposed to interpret such a confession literally and mischievously; and in the second, at no former period has the medical profession been practised with such enlightened views as at the present, and at none shewn itself so disposed to avail itself of every means of augmenting its sphere of usefulness. The increase in the value of human life is progressive: the spirit of investigation is abroad and diffusing: our means of diagnosis are improved and improving; our treatment is more simple, more judicious, and more successful; the excessive use of drugs is, and has long been, much on the decline; while the temptation to its continuance, by making it the means of remuneration, is discountenanced by a very large portion of the profession—and yet this is the period chosen to proclaim aloud the utter rottenness of our system, its want of superiority over a no-system at all, and to declare that, in necessitating its speedy reform, "*the doctrine of Hahnemann will have conferred an inestimable benefit on the healing art!*" Dr. Forbes, it is true, informs us that, with all its faults, the allopathic system of treatment is the best, and that to it he adheres. It is not our business to reconcile or expose these strange inconsistencies on his part, but simply to consider what effect his exaggerated statements must tend to produce upon the profession and upon the public—the interests of both being identical; and we regard this as discouraging and mischievous.

Alarming as his patient's condition may be, Dr. Forbes does not consider it quite desperate, and before taking his leave of him, he furnishes a prescription, consisting of several ingredients, through the instrumentality of which, in the due course of time and nature, relief may yet be hoped for. To our thinking, it is a somewhat expectant affair, and in so serious a case we would fain prefer a more heroic procedure. We are told that we should institute more accurate inquiries into the natural cause and event of diseases, when uninterfered with by art—*upon patients, we hope, who are fully apprised of our intentions:* submit our therapeutical agents to re-examination; endeavour more accurately to ascertain the proportion of curable diseases, and the degree of benefit derivable from medical treatment; and carefully eschew the *post hoc propter hoc* mode of reasoning. For these and other purposes a general adoption of the *Numerical Method* as the means of recording experience is insisted upon. The old maxim, *melius anceps remedium quam nullum,* is to be overturned in favour of Nature's own unmolested operations. Hygiene is pointed out as a fertile field for the medical labourer; and polypharmacy exhibited in colours fitted to make a salutary impression. The Expectant system is elevated at the expense of the Heroic, and active medicines and doses generally discountenanced, especially in exanthemata, fevers, &c., and when given as *placebos.* A greater simplicity in the construction of medicinal formulæ is recommended, and mere routine prescribing held up to animadversion. A more comprehensive and philosophical system of nosology is stated as a desideratum; while professors should shew more anxiety to impart a knowledge of the elements of medical science, than to communicate by rote the names of the medicines which have been found efficacious in various diseases. Endeavours should also be made for enlightening the public mind as to the true powers of medicinal substances, and reconcile them to simpler proceedings—inculcating upon them also the necessity of the early commencement of the treatment of their diseases. "Lastly, and above all, to bring up the medical mind to the standard necessary for studying, comprehending, appreciating, and exercising the most complex and difficult of the arts that are based upon a scientific foundation—the art of Practical

Medicine. And this can only be done by elevating, in a tenfold degree, the preliminary and fundamental education of the Medical Practitioner."

We were desirous of presenting these various suggestions (somewhat pedantically styled "Cogitanda—Excogitanda—Agenda") *in extenso,* but by want of space have been constrained to limit ourselves to an analysis of the most important. Among them we find propositions of undoubted, and others of questionable utility, and very few that can lay any claim to novelty: while, looking at them *en masse,* we cannot but consider that their realization would furnish a very inadequate security for that onward progress which we hope and believe practical medicine is destined to achieve. The assimilation of our practice with that of the Expectant system of medicine, here so strongly counselled, may certainly maintain us upon that footing of equality with our homœopathistic rivals, which the author allows to be our true position, but we doubt whether it will procure for us a continuance of that superiority which we maintain we really possess. Dr. Forbes observes that, since our increased intercourse with the Continent has taught us a greater reliance upon the powers of Nature, we have become more cautious and more patient in the treatment of disease. It is but fair to notice, however, that the influence here spoken of seems to have been reciprocal, for most certainly disease is now treated in France and Germany more actively, and we may add more efficiently, than it was wont to be; and, among the best practitioners of those countries and of our own, the management of the most important affections is now extremely similar. Indeed, we may observe that while, on the one hand, many of the ablest French practitioners resort to doses of powerful medicines of a magnitude unknown to us; on the other hand, those of them who, like M. Magendie, adhere to the strictly expectant treatment, have little enough to boast of as regards the proportion their tedious and imperfect cures bear to the amount of mortality!

We deny that these suggestions can be regarded as the manifesto of "Young Physic." They are rather the offspring of the exaggerated fears and timid previsions of "Old Physic" in his dotage. "Young Physic," not contemning the part of an accurate observer of Nature's operations, sees also a wide sphere of active usefulness, elements of future progress, and good cause for encouragement. He stands forth with his microscope, test-tube, stethoscope, and speculum, intent upon the investigation of the intimate nature and the signs of disease, well knowing that the first step to its more successful treatment is to be found in its more accurate diagnosis. He is aware that an ardent, an enthusiastic spirit of investigation is abroad, which, in the field of Animal Chemistry, has reaped laurels and will gather more; and if the labourers of our own country are fewer than for its honour could be desired, he looks to remedy this in some provision and encouragement for those who may be willing to devote their lives to pursuits of the last importance to humanity, and yet which can never prove remunerative to themselves. Even with the drawback the absence of such must entail, he points with pride to discoveries in anatomy and physiology which have graced our æra, and which have not been barren in practical results. He acknowledges also the extended acquisitions of the present race of medical men as compared even with their immediate predecessors; but, while appreciating the benefit which has and must accrue to the public from the consequent more enlightened practice these enforce, he is not blind to existing defects, and to the necessity of yet far more extensive improvements. For the attainment of these, and various other advantages, he will seek to urge, by every means in his power, the necessity of an Improved Medical Education. To this end he will demand that the present system be entirely reformed, both as regards pupil and teacher. Professional education requires prolongation as well as re-adjustment. The previous years now wasted behind a counter should be employed in storing the mind with facts, disciplining it by science, and enlarging its future sphere of operations by the acquisition of languages. Pursuing his special medical studies, the pupil should be placed under a more active supervision, and submitted to so severe a test by repeated examination, that the trade of the *grinder* might become annihilated. Medical teaching should not be in the hands of those whose length of purse or family influence has raised them to an undeserved pre-eminence; its grave responsibilities being entrusted to those whose talents have been proven by competent judges. It should assume a more practical and less didactical character. Lectures should be essentially demonstrative and clinical; and the student not required to pass hour after hour listening to descriptions of disease which he might more advantageously peruse in his closet, neglecting the while the invaluable opportunities, never again to recur, which surround him, amid the living or the dead, on every side. The crowded condition of the lecture-rooms at some of our medical schools, as contrasted with the deserted state of the hospital-wards, dissecting-rooms, and dead-house, furnish a melancholy subject of contemplation to those who are aware of how little is gained and how much is lost.

These are a few of the objects which may worthily occupy the attention and excite the energies of "Young Physic." If pursued with the earnestness we think there is good prospect of their being, little cause will there be to fear the rivalry of homœopathy, or any other description of quackery, as little to believe in the advent of an impending "Reformation," and still less to doubt of the progressive advancement of legitimate medicine.

Reverting to the two other works upon our list, we have to observe that that by Mr. Leeson is one of abundant pretension and small performance. Professing to exhibit a view of the application of Liebig's Physiology in the Treatment of Disease, he furnishes only indications which were well known, and in approprite cases acted upon, long before that philosopher was born or thought of. Indeed, although he prefaces the practical portion of his work by an imperfect analysis of Liebig's views, with which we should have thought all the world was by this time well acquainted, it has passed our power to discover even an attempt at shewing how these bear upon the management of disease. We can in fact find nothing whatever new in the treatment here recommended, although this is detailed with great self-laudation. The author, it is true, refers to several cases which, condemned by other medical men either as incurable or as requiring operation, were by the persevering use of tonics and abundant food, &c. recovered; but we suppose most of our readers could also refer to such without thinking that they had worked a radical change in medical practice, or endeavouring to elevate such exceptions into a general rule.

Although, perhaps, we are not justified in detaining the reader with a work which, written for a special object, and that a very transparent one, in no-wise deserves his attention, a specimen or two of the author's opinions may at least amuse, if they fail to instruct, him. Speaking of bloodletting, he says:—

"Thousands of cases might have been saved had not bloodletting, and other parts of the antiphlogistic system been employed in the treatment of consumptive cases: on the contrary, had the various outlets through which the system had been allowed to waste been closed, and nutrition carefully introduced, instead of thousands of deaths, we should have thousands of recoveries, and medical science in this department of practice would have been a blessing to mankind, instead of being, as it now is, a horror and a misery. * * * * * While the lungs are undergoing all the changes which constitute the various stages of pulmonary consumption, the stomach frequently remains little impaired; indeed, I might say that I have seen instances where the appetite in many of the advanced stages was almost voracious. It is clear then that, with such advantages in favour of the patient, recovery ought to be looked upon with great hope, if not with certainty, even though the morbid changes in the pulmonary structures had made considerable progress. Almost all the cases which I have had under my care, and where the stethoscope with other means revealed the existence of cavities, and where the integrity of the appetite was yet preserved, got well by putting them under a judicious system of nutrition, combined with such medicines as tend to give vigour to the system and soothe constitutional irritation, and with such pure air as is known to be fit for admission into the respiratory organs." P. 177—180.

The author's experience has been equally successful in other serious maladies, *e. g.*

*Spinal Disease.*—"In every case which came before me, I succeeded in turning the threatened mischief by restoring the constitution to a healthy condition, by means of a well-applied system of nutrition and tonic medicine, together with pure air and moderate exercise." P. 133.

*Mesenteric Disease.*—"I have had an immense number of such cases with children, and when it was my fortune to see a case in time, I made a correction in the dietetic system; and by attending frequently to the secreting and excreting functions, I was enabled to succeed in every such instance." P. 116.

Mr. Leeson is a gentleman of susceptible feelings, as witness.

"*Scrofula.*—I do not like the word scrofula, for it literally means a little sow or pig, and implies a disease to which these animals are subject. Individuals of the highest and most distinguished families in this country have had this disease attributed to them, and often as one of the most delicate, and at the same time one of the most malignant slanders which could be whispered against the interest of honorable and respectable families." P. 115.

They even occasionally betray him into a *little* exaggeration.

"Here we have two cases of disease of the knee-joint condemned to amputation by *pure surgery*, both of which recovered by constitutional treatment alone. Such operations are far from being uncommon in diseases of the knee-joint, and if we are to judge from the two cases which did so well without, how many amputations must have been cruelly and unnecessarily performed by many of the older surgeons. The punishments under surgery, up to a modern date, have been truly awful, and such as our criminal code, in the worst of times, could not have contemplated as punishments for the vilest offences against law and morals." P. 131.

We will conclude our extracts with a curious specimen of the author's pathology.

"Atrophy, or wasting of the lungs, often arises from the absorption of the permanent air in the cell by surrounding inflammation, upon which the mucous surfaces adhere so closely that no re-admission of air within them can be effected." P. 178.

It is refreshing to turn from a book of this description to the one written by Dr. Searle; for although, as regards the latter, we find ourselves unable to agree with either the premises or conclusions of its author, we can testify to the philosophic spirit which has presided over their elaboration, and the valuable character of many of the observations adduced for their elucidation. Would that the principles and practice of physic admitted of the simplification here attempted! Alas! they do not! One point (in addition to another adverted to at the commencement of this article) we decidedly object to; and that is, the author's addressing himself "to the public rather than to the profession," under the delusive hope that, by exhibiting the principles upon which the preservation of health and the removal of disease depend, he is giving a death-blow to the impostures of quacks and deceivers. That the endeavour to inform the public on all those points of hygiene which contribute to the prevention of disease, is a most meritorious (although apparently a very unsuccessful) undertaking, we have repeatedly stated; but we must maintain that, when we come to discuss with it the description and management of disease, we discourse of things it never can and never will comprehend, and by appealing to its judgment and criticism upon subjects so beyond its ken, and which the best-educated even of its members have shewn themselves the most liable to delusions upon, we are sacrificing our own honour, dignity, and means of usefulness, without conferring upon it any equivalent benefit whatever.

Dr. Searle regards electricity evolved from the blood as the actuating principle of life. By its agency the action of the capillary vessels is excited, and the nutrition of the system thus effected. Life is first manifested in these capillaries, and upon the proper maintenance of their due action, its continuance and the preservation of health depend. Therefore "all disease, or derangements of health consist intrinsically and virtually in the disorder or derangement of this, the primary, organic action." The derangements of these vessels may manifest themselves in the form of *congestion* or passive venous plethora of the part affected—*inflammation* or active arterial excitement, and *irritation*, which is not an affection of the nervous system, as usually believed, but consists in a state intermediate between the active condition of the arteries in inflammation, and the passive state of the veins in congestion—and, when general, constituting fever. One of these three states of the capillaries may be assigned as the essential condition of every disease, wherever this may be situated. All disease depending therefore essentially upon the condition of the vascular system, although it may become "modified in character by the nature, structure, and function of the part in which it is centralized, and combinations founded upon the derangements which successively ensue," its treatment becomes much simplified, and is chiefly accomplished through the agency of bloodletting and calomel, the two most powerful instruments we possess for influencing the condition of the capillaries. But blood is not to be taken away in large quantities without discrimination; for, however proper this may be in the case of inflammatory action involving important organs, in the case of congestion, dependent upon debilitating causes, small quantities, slowly abstracted at frequent intervals, are only indicated. Upon the same principle that we relieve the suffering part by reducing the amount of blood circulating in it, we may occasionally effect the same object by exalting the action of the general system by stimuli. In this way do *mercurials* act, independently of their operation on the secretions—of these calomel is the best. Entering into the current of the circulation, it exerts a specific power upon the capillaries—exciting them to increased action. As a general rule, its use in inflammation is limited to the atonic stage, and that of oppression—it being only guardedly given, and in conjunction with other evacuants, in the more active stages.

"The fruits of my experience justify me in declaring, that if there is any single remedy in the cure of disease, meriting the name of *universal*, that remedy is calomel. The explanation I have given of its operation, and the universality of its influence on the system, in exciting the functions of all the organs, and increasing all the secretions, renders it evident, I conceive, that it fulfils indications of one kind or other in the treatment, with few exceptions, of every disease—which are all, it may be truly said, with very few exceptions indeed, based upon the depression of the active energies of life;—health, as I have before said, consisting in the due action and efficient performance of the various functions of the system. Judiciously employed, (Dr. Searle is an advocate for small doses,) I can say with confidence, in opposition to much prejudice on the subject, founded on the circumstances which first introduced it into practice in this country, and its too commonly improper mode of administration, (the principles by which its employment should be regulated not being understood,) that calomel is as harmless as iron, or any other of the articles of daily remedial administration. This conviction, be it remembered, is the fruit of thirty years' experience, twenty of which were spent in India, where this is the chief remedy employed in the cure of disease, and one of universal use both by native and European practitioners." P. 91.

Following out these views, Dr. Searle passes successively to their application in the various diseases to which the frame is liable to. Many of his observations are acute, and some of his practical remarks useful, but we entirely dissent from his view, that a changed condition of the capillary circulation is the sole primary cause of disease. We believe that essential diseases of the nervous system exist and play an important part in disordering the frame; and that remedies widely different from those he has adverted to, are best fitted for the removal of such.

